# Human Decision-Making as a Key Factor in the Risk of Wolf–Dog Interactions during Outdoor Activities

**DOI:** 10.3390/ani11092497

**Published:** 2021-08-25

**Authors:** Andżelika Haidt, Radosław Gawryś, Maciej Szewczyk

**Affiliations:** 1 Department of Forest Ecology, Forest Research Institute, Sękocin Stary, 05-090 Raszyn, Poland; r.gawrys@ibles.waw.pl; 2 Department of Vertebrate Ecology and Zoology, Faculty of Biology, University of Gdańsk, 80-308 Gdańsk, Poland; maciej.szewczyk@ug.edu.pl

**Keywords:** *Canis lupus*, domestic dog, human wildlife conflict, interspecies interactions, behavioral ecology

## Abstract

**Simple Summary:**

The aim of the study was to determine the nature and causes of direct contact between a wolf and domestic dog during different forms of human recreation. The results are crucial for reducing human–nature conflicts and for education. Thanks to this study, we conclude that humans are responsible for reducing the risk of direct contact between these two canine species. The risk of interaction between wolves and a dog that is with a human depends on the distance between the dog and its owner, the number of wolves, and the size of the dog. Hunting with a dog poses a seven times greater risk of interaction with wolves compared to recreational walking.

**Abstract:**

As a result of species protection in Poland, wolves now appear in places that are attractive for human recreation, increasing their exposure to dogs. This creates a risk of spontaneous direct interactions between these two canine species. Aggressive interactions between the gray wolf and the domestic dog lead to human–large predator conflicts. This study examined wolf–dog interactions using data collected in an online questionnaire and included questions related to factors that might influence the likelihood of interactions between these canines. One of the most important factors affecting the likelihood of interaction between a dog and a wolf was the distance between the dog and the human. The number of wolves was also important—the more wolves, the more likely they were to interact with dogs. The risk of interaction also significantly increases with decreasing distance to human settlements. There were also statistical differences in terms of the type of outdoor activity being engaged in. Hunting was seven times more likely to result in a wolf–dog interaction than normal walk. We postulate that the choices made by the human (dog control and type of recreation) caring for the dog are an important factor that can reduce the risk of direct contact between dogs and wolves.

## 1. Introduction

The gray wolf (*Canis lupus*) was previously extirpated from most of Central Europe but has recently recolonized a large part of its historical range [1]. In Poland, the wolf population was severely reduced in the second half of 20th century, when less than a hundred individuals remained, mostly located along the eastern edges of the country [2]. After strict protection across Poland was implemented in 1998, wolves started recolonizing suitable habitats, including vast forest tracts west of the Vistula River [3,4], as well as military training areas [2,5]. In addition, wolves have also been reported in unsuitable and suboptimal habitats [4,6]. The newly formed Central European wolf population is now rapidly expanding westward from its core areas in western Poland and eastern Germany [7], recently reaching Denmark [8], western Germany [9], and Benelux [10,11]. The Carpathian wolf population is also expanding [12]. Thus, the frequency of wolf–dog interactions is expected to increase in most of Central and Western Europe.

Interactions between the gray wolf and the domestic dog take a wide variety of forms, including resource competition, pathogen transfer, and hybridization [13,14]. There are many reasons why dogs are killed by wolves. Wolves often kill dogs, especially hunting dogs, that compete with them for food sources [15]. Domestic dogs may also be killed and used as a food source [16]. Among canid species consumed by wolves, domestic dogs are the most common [17], although ungulates form the main component of the wolf’s diet in Central Europe [18,19,20]. The prominence of dogs in the wolf’s diet is related to the lack of ungulate prey [17]. Predation by wolves on dogs is a source of serious conflict with humans. Domestic dogs are a part of households where they are perceived as members of the family [21]. The increase in the wolf population and their encroachment near large cities leads to an increase in the number of encounters with wolves during human recreational use of green areas. The abundance of natural wolf prey species is lower in areas of higher human density, increasing the likelihood that wolves will include other carnivores in their diet, including domestic dogs [17]. Therefore, it is important to understand the nature of situations where dog interactions with wolves, and in particular dog deaths, occur in order to educate local communities about risks related to wolves, to reduce conflicts between humans and large predators, and to continue to receive public support for efforts to protect wolves.

## 2. Materials and Methods

In this study, a questionnaire method was used to collect data on dog–wolf interactions. The questionnaire was created on the Google platform. The search for respondents took place by querying Facebook’s social network to find thematic groups with subjects related to outdoor recreation with a dog (e.g., “outdoor with dog”, “hunting canine”). Data collection lasted from 5 January 2021 to 19 March 2021. Questions were both open and closed and included: (1) the circumstances of the event (e.g., walking, hunting, and other (such as jogging, bushcraft, search and rescue training, etc.)); (2) geographic location; (3) type of location (forest, road, open habitat); (4) visibility; (5) time of day; (6) year and season; (7) distance between wolf and human; (8) number of wolves observed; (9) wolf behavior, including duration of observation; (10) number of people; (11) number of dogs; (12) size of the dog(s) (based on FCI breeds nomenclature); (13) gender of the dog(s); (14) neutered/spayed; (15) form of dog control (e.g., leash, shock collar); (16) distance between dog and human; (17) was there direct wolf–dog contact; (18) did the owner observe the interaction?; (19) if there was direct contact between animals, which animal initiated it?; and (20) did the interaction result in injury to the dog(s)?

Locations where interactions occurred were mapped and compared with the results of a wolf habitat suitability model [3] and were classified as optimal or sub-optimal habitats according to the model.

### 2.1. Ethical Statement

All interviewed persons gave consent for the use of provided data on wolf behavior. No minors were interviewed.

### 2.2. Statistical Analysis

All calculations were performed using the program R [22]. To determine the relationship between the occurrence of wolf–dog interactions and explanatory variables collected in the questionnaire, logistic regression was employed using GLMs (generalized linear models) with a binomial distribution and the log link function. For this purpose, the “glm” function from the R Stats Package [22] was used, first analyzing the influence of single explanatory variables on the interaction. Then, all explanatory variables for which the *p*-value of the Wald test from the single model was not greater than 0.05 were used in a joint model. To achieve the best fit for the model, the “step” function was used, which performed multiple comparisons of different combinations of the given variables to achieve the lowest possible AIC (Akaike Information Criterion) value. The variables used in the analysis were checked for the presence of collinearities by calculating the Variance Inflation Factor using the “vif” function from the “car” package in R [23]. If VIF was 3, variables were considered collinear. To illustrate relationships between variables used in the full model, PCA (Principal Component Analysis) was performed in the “vegan” package [24]. Variables used were standardized using the “decostand” function (method = “standardize”). Qualitative variables were used in binary form (0, 1). Spearman’s rank correlation coefficient was calculated using the “cor.test” function in the “stats” package. Results were considered statistically significant at *p* < 0.05.

## 3. Results

**Figure 1:**
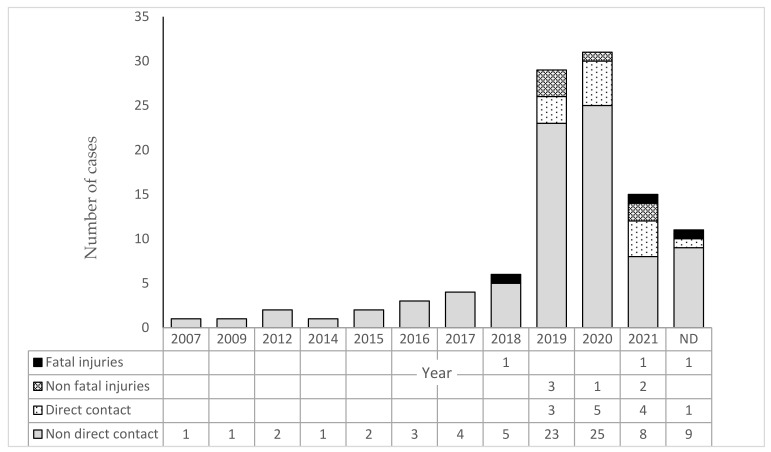
Number of wolves observed during outdoor activities with dogs and the number of observations that ended in direct wolf–dog contact, including those with fatal and non-fatal injuries.

We analyzed 106 cases of wolf sightings during outdoor recreation with a dog. Direct wolf–dog contact occurred in only 21% of cases. Dogs were injured in 8% cases, with fatal injury in 3% of cases (Figure 1). There was a statistically significant increase in the number of wolf sightings reported over the period 2007–2020 (rho = 0.95; *p* < 0.001). The power of individual variables in the GLM model to explain the occurrence of direct contact during wolf–dog encounters is shown in Table 1 and Table 2. Contact resulting in injury occurred at a distance of 0.05–1 (Med = 0.175) km from buildings. Dog injuries occurred when wolves were 15–300 (25) meters from the human and when dogs were 0.5–100 (22.5) meters from the human. Injuries to dogs occurred when there were 1–10 (4) wolves, 1–5 (2.5) humans, and 1–5 (2) dogs present. Wolves were observed from 0–120 s prior to an attack taking place. In the three cases where there was a fatal attack on a dog, they occurred within 0.3, 1, and 2 km of buildings, the wolf was 15, 500, and 30 m from the human, and the dog was 400, 500, and 20 m from the human. There were: 1 and 7 wolves, 3, 1, and 3 humans and 3, 1, and 4 dogs involved in fatal incidents. The period of time the wolf was seen before an attack on a dog occurred was 30, 0, and 5 s. In two of the three fatal encounters, humans and dogs were involved in hunting. One of the fatalities involved a walk with the dog 500 m from its owner.

During 2007–2021 (2021 covering only the first quarter of the year), there were statistically significant increases in the mean observed distance between wolf and human (rho = 0.64, *p* = 0.040) and in the mean number of wolves sighted (rho = 0.62, *p* = 0.042). A positive relationship (rho = 0.24, *p* = 0.012) was also shown between the length of time a wolf was observed and the distance between wolf and human.

**Figure 2:**
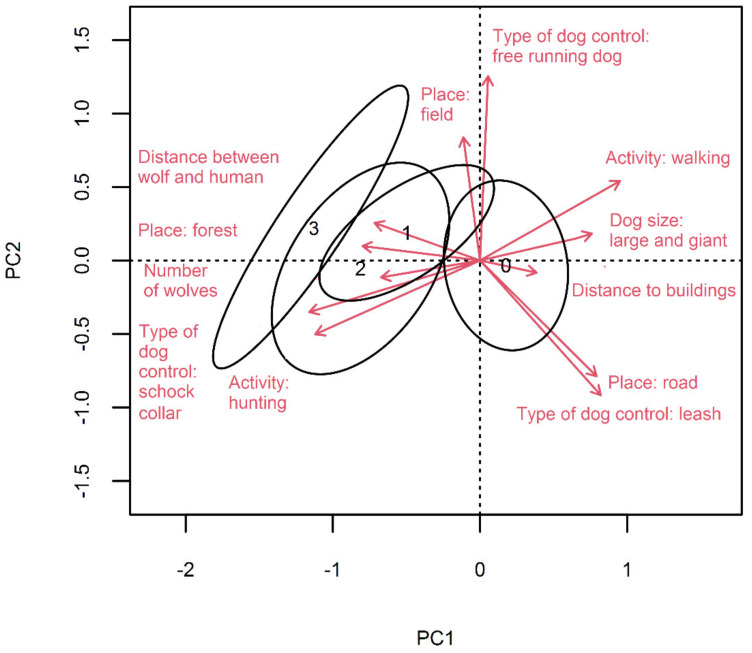
Biplot of principal component analysis based on the variables (arrows) that significantly influence dog–wolf interactions according to GLM analysis. Observations were grouped according to the degree of interaction (0 – no interaction, 1 –interaction without injuries, 2 – interaction with non-fatal injuries, 3 – interaction with fatal injuries) and presented as an ellipse (1SD from the centroid of the observation cloud). For categorical variables, each category is presented as a separate vector. The PCA1 axis accounted for 27.9% of the variance, PCA2 for 17.7%.

The variables from Table 1 and Table 2, which according to the GLM model had a significant influence on the occurrence of wolf–dog interactions (distance to buildings, distance between dog and human, number of wolves, size of the dog, activity, place, type control over the dog), are shown in the PCA diagram (Figure 2). This diagram also groups observations by degree of interaction. Both the number of dog–wolf interactions and the likelihood of interactions resulting in dog injury or death are positively correlated with the occurrence of an incident within the forest (i.e., further from roads), the distance between dog and human, the number of wolves, whether humans were hunting, and type of control being shock collar. On the other hand, an increase in the distance to buildings, the presence of large dogs, and if the human activity was walking were associated with reduced likelihood of dog injury or death. It is noteworthy that hunting and walking produced opposite effects on interactions with wolves, as did distance from buildings. Walking a dog on a forest trail was strongly correlated with the use of a leash and was associated with a lower risk of wolf interactions.

Using the results from the GLM model for single explanatory variables, variables that had a significant influence on the occurrence of wolf–dog interaction were used to build a multi-factor model, as follows:

GLM (interaction ~ Distance to buildings (km) + Number of wolves + Distance between dog and human (m) + Activity + Place + Dog size + Type of dog control, family = binomial).

The Akaike information criterion (AIC) obtained for this model was 76.8. After applying the “step” function, as a result of which the variables “Activity” and “Dog size” were discarded, the following model was obtained:

GLM (interaction ~ Distance to buildings (km) + Number of wolves + Distance between dog and human (m) + Place + Type of dog control, family = binomial), details of which are presented in Table 3. The AIC of the reduced model was 73.0. No collinearities were found in either model (VIF < 3).

## 4. Discussion

This study documents the increasing incidence of wolf sightings during human recreation and direct contact between wolves and domestic dogs in recent years. While it is possible that some events from more than a decade ago have been forgotten and not been reported in the survey, the fact that wolf sightings are strongly memorable events, especially when they result in interactions with a family dog, makes a lack of recall less likely. An important factor analyzed in the survey was whether the size of the dog affected the incidence of wolf–dog contact. Wolves may perceive larger dogs as a physical threat, making wolves less likely to initiate close contact. For this reason, livestock guardian dogs, for example, are a large breed of dog that are very effective at defending livestock and themselves against wolves [25,26] and present a risk of injury to the wolf. A risk of injury to the wolf is likely to reduce contact with potentially dangerous large dogs [27].

There was a statistically significant increase in recent years in both the likelihood of direct contact with wolves and with increasing sightings of larger family groups of wolves. It is probable that when wolves outnumber a dog that the wolves are less likely to be injured, even in confrontations with larger dogs. Injuries to a predator can reduce its ability to hunt for food and survive [28]. Another factor that may explain wolf aggression towards dogs is territorialism. Sedentary packs are more likely to defend their territories and attack intruders, while lone individuals are often dispersers/floaters that use a given area transiently and thus are less likely to defend it. Moreover, large family groups of wolves are expected to be bolder, as is seen in cases of intraspecific aggression, where packs with more adult members are more likely to be successful in a conflict [29]. 

Another significant finding is that hunting increased the risk of dog–wolf interactions sevenfold. This suggests that specific types of human behavior shape the behavioral response of wolves to domestic dogs. Hunters generally choose areas with the greatest concentration of game, as high ungulate densities result in financial losses to forestry and agriculture. Wolves usually hunt where game is most plentiful [30]. Therefore, hunting dogs are perceived by wolves as competition for food resources [31,32] and wildlife predation by dogs is a serious conservation concern [33]. Hunting dogs are also at greater risk of encountering large groups of wolves, as hunters more often prefer to use the centers of large forests that are core wolf territories. 

Many species of animals show strong anthropophobia [34,35]. The gray wolf is one such species, which was confirmed by the results of this study. The further a dog is from humans, the greater the risk of direct contact between wolf and dog. It can therefore be assumed that human presence provides a protective shield against wolves.

A very interesting result of this study is the increased risk of interaction between wolves and dogs nearer to buildings. The closer to buildings, the greater the likelihood of a wolf–dog interaction. While in general wolves avoid human buildings [36], at times they appear in the vicinity of such structures. Why might wolves be found near human buildings? The first possible answer is for food, such as hunting livestock or predation of a dog. Wolf predation is a cause of domestic animal mortality [37,38,39,40]. The second possibility is that wolves are found near buildings transiently during dispersion, especially in winter and spring [41,42]. Anthropopressure is associated with an increase in stress hormones in wild animals [43]. There is also a strong association between stress hormones and aggressive responses in animals [44]. Buildings and the infrastructure associated with buildings limit the possibility of escape and a feeling of being “cornered” can increase the risk of animal attack. On the other hand, hunger and the desire to obtain easily accessible prey in the form of livestock are associated with the activation of the canine prey drive, which is also linked with increased cortisol level. Another possible explanation for the different reactions of wolves depending on the distance from the building is habituation. Animals that are habituated to humans behave differently than animals that are not habituated to humans [45]. Our research has shown that one of the most important factors influencing wolves’ behavior toward dogs is their fear of humans. Habituated wolves that are less fearful of humans can be more likely to come into direct contact with domestic dogs.

It should be emphasized that not every direct wolf–dog contact ends in attack leading to the injury or death of the dog. The disadvantage of the current survey is that it does not give information about the age, sex, and reproductive status of the wolves encountered. It should be noted that cases of aggression between dogs most often involve individuals of the same sex (male–male and female–female) [46]. Note that we managed to collect a relatively low number of interactions with which to look for trends/patterns. Finally, the questionnaires were completed by volunteers whose education was not necessarily related to the natural sciences. Therefore, there is a risk of incorrect species identification and confusion of wolves with certain dog breeds, e.g., husky or wolfhound. Despite the risk of errors in data collection, we believe the findings of this study provide valuable information for the conservation of wolves in a human-dominated world.

## 5. Conclusions

Human presence is an important factor affecting whether direct contact occurs between wolves and domestic dogs. The greater the distance between the dog and its owner and the greater the number of wolves, the greater the risk of direct contact between dog and wolf. For this reason, dog walking is much less risky for dogs compared to hunting, as the distance between human and dog is usually small and the human is a shield for their dog. Human decisions regarding the type of activity and control of the dog are significant. Walking a dog on a forest trail should be done with the dog leashed, as it keeps the dog close and thereby reduces the likelihood of a wolf–dog interaction. This study indicates that educating dog owners about risk reduction approaches can provide crucial information that protects their dogs and reduces the conflict between man and wildlife.

## Figures and Tables

**Table 1:** Descriptive statistics of continuous variables and GLM (family = binomial) results of parameters estimated to explain occurrences of dog–wolf direct contact (models with one explanatory variable).

Variables	Direct Contact		GLM = Interaction~Variable						
	No (*n* = 84)	Yes (*n* = 22)							
	min, med, max	min, med, max	Intercept	SE	*p*	Estimator	SE	*p*	AIC
Distance to buildings (km)	0.01; 2; 10	0.02; 0.5; 3	−0.440	0.362		−0.651	0.249	**	101.7
Distance between wolf and human (m)	5; 55; 300	2; 30; 500	−1.361	0.338	***	0.000	0.003		112.26
Distance between dog and human (m)	1; 2; 500	0.5; 50; 500	−1.738	0.287	***	0.009	0.003	***	99.583
Number of wolves	1; 1; 6	1; 2; 10	−2.263	0.419	***	0.381	0.128	**	102.59
Number of humans	1; 1; 15	1; 1; 20	−1.550	0.301	***	0.097	0.080		110.83
Number of dogs	1; 1; 10	1; 1; 5	−1.437	0.366	***	0.051	0.144		112.14
Observation (s)	0; 9; 900	0; 0; 300	−1.306	0.257	***	−0.002	0.003		107.72

** *p* < 0.01; *** *p* < 0.001.

**Table 2:** Descriptive statistics of binary and ranked data and GLM (family = binomial) results of parameters estimated to explain the occurrence of dog–wolf interactions (models with one explanatory variable).

Variable	Level	Direct Contact		Effect of Direct Contact		GLM = Direct Contact~Variable			
		No	Yes	Injury	Fatal Injury	Estimator	SE	*p*	AIC
Habitat	Not optimal	22	5	2		−1.482	0.495	**	112.2
	Optimal	62	17	4	3	0.188	0.566		
Visibility	≤50 m	19	4	2		−1.558	0.550	**	112.1
	>50 m	65	18	4	3	0.274	0.611		
Neutered/spayed dog	No	52	17	5	3	−1.118	0.279	***	110.4
	Yes	32	5	1		−0.738	0.556		
Contact with wolf	No	79	20	5	3	−1.374	0.250	***	112.0
	Yes	5	2	1		0.457	0.873		
Winter	No	47	7	3		−1.904	0.405	***	104.3
	Yes	35	14	3	2	0.988	0.514		
	Nd	2	1		1				
Time of day	No	25	6	2	1	−1.427	0.455	**	112.2
	Yes	59	16	4	2	0.122	0.535		
Dog size	Small and medium	39	17	5	3	−0.830	0.291	**	105.3
	Large and giant	45	5	1		−1.367	0.554	*	
Activity	Walking	59	7	1	1	−2.132	0.400	***	101.1
	Other	11	3			0.832	0.764		
	Hunting	14	12	5	2	1.978	0.561	***	
Place	Forest road	52	3	1		−2.853	0.594	***	95.4
	Forest	15	12	3	2	2.630	0.709	***	
	Field	17	7	2	1	1.965	0.745	**	
Dog gender	Male	34	13	5	2	−0.961	0.326	**	111.3
	Female	33	7	1	1	−0.589	0.529		
	Male and female	17	2			−1.179	0.816		
Type of dog control	Leash	50	3	1		−2.813	0.594	*	90.7
	Shock collar	7	11	3	2	3.265	0.766	**	
	Free running dog	27	8	2	1	1.597	0.718	**	

* *p* < 0.05; ** *p* < 0.01; *** *p* < 0.001.

**Table 3:** Average parameter estimates for binomial GLM explaining the occurrence of dog–wolf interactions (multiple variable model) AIC: 73.0.

Variables	Estimate	SE	*p*	Exponentiated Coefficients
Intercept	−3.278	1.006	0.001	0.038
Distance to buildings (km)	−1.203	0.416	0.004	0.300
Distance between dog and human (m)	0.007	0.003	0.053	1.007
Number of wolves	0.348	0.226	0.124	1.416
Place: Forest (vs. Road)	2.834	1.006	0.005	17.018
Place: Open habitat (vs. Road))	1.300	0.896	0.146	3.671
Type of dog control: (Shock collar vs. leash)	2.324	1.114	0.037	10.212
Type of dog control: (Free running dog vs. leashed)	1.252	0.828	0.131	3.496

## Data Availability

The data presented in this study are available on request from the corresponding author.
